# Effective Use of microRNA, BRAF and Sonographic Risk Assessment in Bethesda III Thyroid Nodules Requires a Different Approach to Nodules with Features of Nuclear Atypia and Other Types of Atypia

**DOI:** 10.3390/cancers15174287

**Published:** 2023-08-27

**Authors:** Dorota Słowińska-Klencka, Bożena Popowicz, Dominika Kulczycka-Wojdala, Bożena Szymańska, Joanna Duda-Szymańska, Martyna Wojtaszek-Nowicka, Krzysztof Kaczka, Mariusz Klencki

**Affiliations:** 1 Department of Morphometry of Endocrine Glands, Medical University of Lodz, Pomorska 251 St., 92-213 Lodz, Poland; dsk@tyreo.umed.lodz.pl (D.S.-K.); bozena.popowicz@umed.lodz.pl (B.P.); 2 Research Laboratory CoreLab, Medical University of Lodz, Mazowiecka 6/8 St., 92-215 Lodz, Poland; dominika.kulczycka-wojdala@umed.lodz.pl (D.K.-W.); bozena.szymanska@umed.lodz.pl (B.S.); 3 Department of Pathomorphology, Medical University of Lodz, Pomorska 251 St., 92-213 Lodz, Poland; joanna.duda-szymanska@umed.lodz.pl; 4 Department of Clinical Endocrinology, Medical University of Lodz, Pomorska 251 St., 92-213 Lodz, Poland; martyna.wojtaszek-nowicka@umed.lodz.pl; 5 Department of General and Oncological Surgery, Surgical Clinical Sciences, Medical University of Lodz, Pomorska 251 St., 92-213 Lodz, Poland; krzysztof.kaczka@umed.lodz.pl

**Keywords:** thyroid cancer, FNA, EU-TIRADS, BRAF, microRNA, AUS-nuclear, AUS-other

## Abstract

**Simple Summary:**

Commercially available molecular tests for thyroid fine needle aspiration biopsy are expensive and usually require an additional aspiration of the nodule. Therefore, we checked whether a method free of these main disadvantages would be feasible in the case of Bethesda category III nodules. To this end, we decided to use the material remaining in the needle after preparation of the classic smear and to limit molecular diagnostics to a few well-recognized tests: BRAF V600E mutation and three microRNAs, miR-146b, miR-221 and miR-222. At the same time, we assessed the potential of the combined ultrasound and molecular evaluation of nodules and considered the distinctiveness of two main subcategories of Bethesda category III: nodules with nuclear atypia and nodules with other types of atypia (predominantly architectural). As we show, this approach facilitates clinical decision making for both of these subcategories and prevents a significant number of diagnostic thyroid surgeries.

**Abstract:**

The aim of the study was to analyze the diagnostic usefulness of the combined assessment of the ultrasound risk category of the nodule (evaluated with EU-TIRADS system), the presence of BRAF V600E mutation and the expression of selected microRNAs (miR-146b, miR-221 and miR-222) in Bethesda category III thyroid nodules, separately for cases with nuclear atypia (AUS-nuclear) and cases with other types of atypia (AUS-other). We evaluated 161 nodules (66 AUS-nuclear and 95 AUS-other) with known results of postoperative histopathological examination. The rate of cancer and the rate of PTC among cancers were nearly three times higher in the AUS-nuclear than the AUS-other group. For AUS-nuclear nodules, the most effective diagnostic panel included, in addition to repeat FNA, the assessment of BRAF V600E mutation and the expression of miR-146b and miR-222 (sensitivity: 93.5%, specificity: 80.0%). For AUS-other nodules, a two-step procedure was most effective: at the first stage, forgoing surgical treatment in subjects with a benign repeat FNA outcome, and, at the second stage, the assessment of miR-222 expression and the EU-TIRADS category (sensitivity: 92.3%, specificity: 76.8%). The optimal use of molecular methods in the diagnostics of category III thyroid nodules requires a separate approach for nodules with nuclear atypia and nodules with other types of atypia.

## 1. Introduction

Fine needle aspiration biopsy (FNA) is the primary test used in the diagnosis of thyroid nodules. Its outcome determines the need for surgical treatment or allows for a conservative approach [1,2]. Unfortunately, three out of six categories in the commonly used Bethesda classification of thyroid cytology outcomes are indeterminate, i.e., categories III, IV and V [3,4,5]. Among these categories, category III is the most controversial, because, due to its heterogeneity, it presents a strongly variable risk of malignancy between centers, ranging from a few to even 70% [6,7,8,9,10]. This variability is a consequence of not only epidemiological differences but also the ununiformed interpretation of imprecise rules for the diagnosis of this category. Currently, there are two main subcategories usually distinguished within category III: the subcategory with nuclear atypia and the subcategory with other types of atypia, particularly those characterized by architectural atypia. The former is commonly referred to as ‘AUS’ (atypia of undetermined significance) and it may entail even a two-times higher risk of malignancy than the latter subcategory, called ‘FLUS’ (follicular lesion of undetermined significance) [8,9,10,11]. Since this year’s update of the Bethesda classification recommends the term “AUS-nuclear” for cases with nuclear atypia and the term “AUS-other” for other nodules of category III, we adopt this terminology in the current study, instead of ‘AUS’ and ‘FLUS’, respectively [5]. If a malignant neoplasm is diagnosed in a nodule of category III, it is a papillary thyroid carcinoma (PTC) in the vast majority of AUS-nuclear nodules, while malignancies observed in AUS-other nodules are more diverse and quite often include also follicular thyroid carcinoma (FTC) [7]. These carcinomas differ not only in their microscopic images but also in their ultrasound images and molecular background [1,2,12,13,14,15]. This is important because it is recommended to perform a repeat FNA (rFNA) of nodules classified as category III, along with an assessment of the risk of their malignancy based on the selected ultrasound risk stratification system and, if possible, also a molecular examination of cytological material [1,2,16]. However, publications on the effectiveness of such examinations usually do not consider AUS-nuclear and AUS-other nodules separately. This is probably one of the most important factors causing the lack of consistent results of such analyses.

There are several molecular tests currently available for the diagnostics of thyroid nodules with indeterminate cytology, such as a gene expression classifier (GEC), gene mutational panel, targeted next-generation sequencing (tNGS) and the combination of mutation detection and microRNAs (miRNAs) expression [14,15,16,17,18,19,20,21,22,23,24]. Their application is limited by their high costs. Moreover, many of these tests rely on a dedicated pass of FNA. To avoid this need, attempts are made to use FNA residual material that contains cells remaining in the needle after smear preparation. This material can be preserved during the first FNA and used in the case of an inconclusive cytology result. Despite its limited amount, in some centers, such material is routinely used to assess the presence of BRAF mutation—characteristic of PTC [16]. In the case of a positive result, it is even possible to forgo rFNA. Unfortunately, with almost 100% specificity, BRAF mutation assessment has, with the exception of East Asian countries, unsatisfactory sensitivity [16]. Other attempts to use the biopsy residual material involve the evaluation of microRNA (miRNA) expression. These small RNA molecules play a major role in the posttranscriptional regulation of gene expression, and their deregulation is associated with cancer development and aggressiveness [25,26,27,28]. However, studies dedicated to assessing the usefulness of miRNAs, as with other molecular analyses, are usually not performed separately for AUS-nuclear and AUS-other nodules. Such studies do not take into account the ultrasound image of the nodule either, although it is routinely assessed in clinical decision making in patients with indeterminate cytology.

Therefore, the aim of our study was to analyze the diagnostic usefulness of the combined assessment of the ultrasound risk category of the nodule, the presence of BRAF mutation in its cells and the expression of selected miRNAs in the residual material from routine FNA separately for AUS-nuclear and AUS-other nodules. The ultrasound risk was assessed with the EU-TIRADS system (Thyroid Imaging Reporting and Data System), which is recommended by the European Thyroid Association (ETA) and is routinely used in our center [1]. Previous reports, including ours, indicate that this system is distinguished by its effectiveness in the case of nodules with equivocal cytology [29,30]. Three different miRNAs, miR-146b, -221 and -222, were selected for evaluation on the basis of a literature review, for which overexpression was confirmed primarily in PTCs but also in FTCs as compared to their levels in normal thyroid tissue [25,26,27,28,31,32].

## 2. Materials and Methods

### 2.1. Examined Patients

The study was performed in years 2020–2023 in a single center, in which FNA of the thyroid had been performed for many years in patients referred from an outpatient clinic of endocrinology. The selection of nodules for FNA was generally made according to the recommendations of the ETA, following the categorization of the nodule into an appropriate EU-TIRADS category [1]. In some cases, due to the preferences of the patient or the referring doctor, FNA was performed in nodules smaller than the recommended size for each EU-TIRADS category. The biopsy was performed under ultrasound guidance and consisted of two aspirations of the selected nodule. Smears were fixed with 95% ethanol solution and stained with hematoxylin and eosin. FNA outcomes were formulated according to the Bethesda System for Reporting Thyroid Cytopathology (BSRTC) [3,4]. All nodules classified as category III of BSRTC were assigned to one of its two main subcategories: cases with nuclear atypia (AUS-nuclear) and cases with other types of atypia, particularly those characterized by architectural atypia alone or a predominance of oncocytes or atypia not otherwise specified (AUS-other) [5]. The diagnosis of AUS-nuclear was made in cases with the presence of local features suggestive of PTC (nuclear grooves, enlarged nuclei with pale chromatin and alterations in nuclear contour and shape) in an aspirate that was otherwise benign in microscopic appearance or for specimens with limited cellularity but with nuclear atypia. The diagnosis of AUS-other was made primarily when the specimen showed features on the borders of categories II and IV, especially some degree of architectural atypia (microfollicles, trabeculae or crowding), but insufficient for the diagnosis of neoplasia.

Patients with FNA outcomes of BSRTC category III were—following the guidelines—routinely referred to repeat FNA, which was performed 3 to 12 months after the first FNA. During rFNA, the material was secured for molecular procedures by flushing a biopsy needle with a dedicated buffer. The material obtained in this way was placed in a low-temperature freezer until the planned molecular tests were carried out.

The molecular assessment was performed in patients with AUS-nuclear and AUS-other nodules with a known final postoperative histopathological diagnosis. This was the case in 152 patients with 163 nodules. Patients were referred for surgical treatment by the doctors of the endocrine outpatient clinic. The decision regarding surgical treatment was made based on the results of routine examinations and patient preferences (without molecular tests). The histopathologic examination was performed according to the standard procedure and its results were formulated according to the WHO classification of thyroid tumors [33,34]. For the sake of the study, follicular cell-derived low-risk neoplasms (non-invasive follicular thyroid neoplasm with papillary-like nuclear features—NIFTP; thyroid tumors of uncertain malignant potential and hyalinizing trabecular tumors) were included in the group of malignant nodules. According to the assumptions adopted by the WHO, these neoplasms are borderline tumors that are morphologically and clinically intermediate between benign and malignant tumors but with the potential (extremely low) to develop metastasis [12]. In the case of 2 nodules, insufficient material was obtained during isolation. Eventually, molecular tests were performed in material obtained from 161 nodules, including 66 AUS-nuclear nodules and 95 AUS-other nodules. There were 44 cancers revealed among them (Table 1). None of the persons involved in performing the molecular tests had any information on the FNA results, EU-TIRADS category or postoperative histopathological findings.

### 2.2. Extraction of Total RNA and Genomic DNA

Genomic DNA and total RNA were isolated from residual FNA samples using the AllPrep DNA/RNA Mini Kit (Qiagen, Hilden, Germany). Residual material, flushed from the aspiration needle, was collected in 2 mL Eppendorf tubes with 500 µL of RNAlaterTM Stabilization Solution (ThermoFisher Scientific, Waltham, MA, USA) and stored at −80 °C. After thawing, samples were centrifuged for 10 min at 3000× *g*, and then the supernatant was removed and the pellet was pooled in 300 µL of RTL Plus Buffer (Qiagen) with 3 µL of β-mercaptoethanol. In the next step, samples were homogenized using TissueRuptor (Qiagen) and an additional 300 µL of RTL Plus Buffer with 3 µL of β-mercaptoethanol was added. The further procedure was performed according to the manufacturer’s protocol.

Briefly, the homogenate was loaded onto the DNA binding column and the filtrate was used to isolate the total RNA, including miRNA. Then, the filtrate was digested with proteinase K, 100% ethanol was added and the mixture was incubated at room temperature. After loading on the RNA binding column, it was washed with RPE buffer and digested with DNAse. The next step consisted of washing the column several times with dedicated buffers from the kit and eluting the RNA with 30 µL of RNase-free water. The purified total RNA was immediately used for cDNA synthesis or stored at −80 °C.

The column containing DNA was washed with AW1 buffer and treated with proteinase K to eliminate residual proteins. Then, the column was washed with dedicated buffers from the kit and DNA was eluted with 50 µL EB buffer. The DNA was stored at −20 °C until use.

### 2.3. Detection of BRAF Mutation

Detection of BRAF V600E mutation by ddPCR was performed using 11 μL ddPCR Supermix for Probes (no dUTP, Bio-Rad, Hercules, CA, USA), 1.1 μL ddPCR Mutant Assay BRAF V600E (Bio-Rad) and 9.9 μL (maximum volume) of DNA. Water was used as a ‘no template control’ (NTC) and DNA from a PTC as a positive control.

Droplet digital PCR assays were carried out in a final volume of 20 μl reaction mixture. Droplets were created using the QX200 Droplet Generator (Bio-Rad) and DG8 disposable cartridges. Then, 70 μL of droplet generation oil was placed into the oil well for each sample. The amplification was performed using the GeneAmp PCR System 7900 (Applied Biosystems, Waltham, MA, USA) at the following conditions: 1 cycle of 95 °C for 10 min, 40 cycles of 94 °C for 30 s and 55 °C for 1 min (ramp rate 2.5 °C/s), 1 cycle of 98 °C for 10 min and a 4 °C hold. Analysis of the ddPCR data was performed with the QuantaSoft analysis software, version 1.7.4.0917. Only results with a number of droplets > 10,000 were included in the analysis.

### 2.4. miRNA Expression

The quantity and quality of total RNA was measured with a PicoDrop spectrophotometer (Picodrop Ltd., London, UK). When the concentration of RNA obtained during the isolation process was below 2 µg/mL, 5 µL of RNA eluate was used for the reverse transcription reaction. Reverse transcription was carried out with 10 ng of the total RNA in 15 μL of reaction mixture using the TaqMan^®^ MicroRNA Reverse Transcription Kit (Applied Biosystems), according to the manufacturer’s instructions. MicroRNA quantification was done using standard TaqMan^®^ MicroRNA Assays (Applied Biosystems): hsa-miR-146b (assay ID 474220), hsa-miR-221 (assay ID 000524), hsa-miR-222 (assay ID 002276) and hsa-miR-625* (assay ID 002432) as an endogenous control. Here, 20 μL of the qPCR reaction mixture included 1.33 μL of RT product, 10 μL of TaqMan Fast PCR Master Mix and 1 μL of TaqMan miRNA Assay (20x). The mixtures were incubated in a 96-well plate at 95 °C for 3 min, followed by 40 cycles of 95 °C for 5 s and 60 °C for 20 s. All reactions were run in duplicate or—when the difference in Cq values was larger than 0.5—in quadruplicate.

TaqMan PCR assays were performed on a 7900HT Fast Real-Time PCR System (Thermo Fisher Scientific) and analyzed using SDS 2.3 and the DataAssist software version 3.01 (Applied Biosystems). Fold change values were calculated according to the ΔΔCt method.

### 2.5. Assessment of EU-TIRADS Category

Each FNA was preceded by the ultrasound imaging of the neck. All ultrasound examinations were performed with the use of the Aplio α ultrasound system (Canon Medical Systems Europe B.V., Zoetermeer, The Netherlands) with a matrix linear probe, 18LX7. An analysis of US malignancy risk features was carried out prospectively and directly preceded FNA. Particular sonographic features relevant to EU-TIRADS were identified by experienced sonographers (doctors with a minimum of ten years of experience), according to a unified pattern that was described in detail in our previous reports. In this way, all thyroid nodules were classified into specific categories of EU-TIRADS [1]. The system comprises 5 categories. EU-TIRADS 1 denotes a US examination with no thyroid nodules found. EU-TIRADS 2 (benign category) comprises two different patterns: pure/anechoic cysts and entirely spongiform nodules. EU-TIRADS 3 (low-risk category) comprises isoechoic or hyperechoic nodules with an oval shape, smooth margins and without any feature of high risk of malignancy. EU-TIRADS 4 (intermediate-risk category) includes mildly hypoechoic nodules of an oval shape, with smooth margins and without any feature of high risk. In the case of the heterogeneous echogenicity of the solid component, the presence of any hypoechoic part classifies the nodule as intermediate risk. EU-TIRADS 5 (high-risk category) comprises nodules with at least 1 of the following high-risk features: non-oval shape, irregular margins, microcalcifications and marked hypoechogenicity.

### 2.6. Data Analysis and Statistical Evaluation

In the first step, the diagnostic efficacy of each FNA auxiliary method was analyzed separately for AUS-nuclear and AUS-other nodules. To this end, the incidence of BRAF mutation and the EU-TIRADS categories, as well as fold change values indicating the expression of examined miRNAs, were compared with respect to the division of the AUS-nuclear and AUS-other nodules into benign lesions and cancers. Next, the threshold category of EU-TIRADS and cut-off values of miRNA expression were identified by the evaluation of the Youden index of the receiver operating characteristics curve (ROC). The effectiveness of the determined thresholds and of BRAF mutation presence was shown in terms of the sensitivity (SEN), the specificity (SPC), the accuracy (ACC), the positive predictive value (PPV), the negative predictive value (NPV), the positive likelihood ratio (LR+) and the percentage of nodules reaching the given cut-off value. We used logistic regression analysis to determine the odds ratio (OR) with relative 95% confidence intervals (95% CI) for the established thresholds. The effectiveness of all examined additional methods (EU-TIRADS, BRAF mutation and miRNAs) was compared with their area-under-the-ROC (AUC) values. The usefulness of all methods in revealing cancers with negative results of rFNA was also determined. Results of rFNA were considered positive if they were of category V or VI BSRTC.

In the second step, the diagnostic efficacy of all possible combinations of EU-TIRADS, BRAF and miRNA methods was evaluated. The results were presented as SEN, SPC, ACC, PPV, NPV, LR+ and the percentage of nodules that satisfied particular joint criteria.

The statistical analysis was conducted with the use of Dell Statistica (data analysis software system), version 13, Dell Inc. (2016), Round Rock, TX, USA. The comparison of frequency distributions was performed using the chi-square test (with modifications appropriate for the number of analyzed cases). Continuous variables were compared between groups with the use of the Kruskal–Wallis or Mann–Whitney test. The AUC values were compared with the Z test. The value of 0.05 was assumed as the level of significance. The study design was approved by the Local Bioethics Committee. The approval code is “RNN/26/20KE” (14.01.2020). All patients gave informed consent.

## 3. Results

Malignant neoplasms were eventually diagnosed in 47.0% of AUS-nuclear nodules and 13.7% of AUS-other nodules (*p* < 0.0001) (Table 1). The rate of PTC among cancers in AUS-nuclear nodules was nearly three-times higher than in the group of AUS-other nodules (87.1% vs. 30.8%, *p* = 0.0007). Apart from PTCs, the malignancies found in AUS-nuclear nodules included one FTC, one NIFTP, one hyalinizing trabecular tumor and one squamous cell carcinoma, while, in AUS-other nodules, there were three FTCs, three thyroid tumors of uncertain malignant potential, one medullary carcinoma, one malignant lymphoma and one secondary tumor (metastasis of clear cell renal carcinoma).

### 3.1. Effectiveness of EU-TIRADS Categorization, BRAF Mutation and miRNA Assessment in AUS-Nuclear and AUS-Other Nodules

Malignant nodules of both the AUS-nuclear and AUS-other type were classified as EU-TIRADS category 5 more often than their benign counterparts (AUS-nuclear: 58.1% vs. 11.4%, *p* = 0.0001; AUS-other: 46.2% vs. 8.5%, *p* = 0.0002) (Table S1). The same EU-TIRADS category 5 was the best cut-off value for both AUS-nuclear and AUS-other nodules, which showed the highest sum of SEN and SPC and the highest ACC in the differentiation of malignant nodules from benign ones. The classification of a nodule as category 5 significantly increased its risk of malignancy regardless of its type (OR, AUS-nuclear: 10.7; AUS-other: 9.2) (Table 2 and Table 3).

The BRAF mutation was revealed in 20 (64.5%) malignant AUS-nuclear nodules and one (7.7%) malignant AUS-other nodule, all of which corresponded to PTCs. For AUS-nuclear nodules, BRAF-mutated PTCs accounted for 74.1% (20 out of 27) of all PTCs, and for AUS-other nodules, they accounted for 25.0% (1 out of 4) of all PTCs. There was no case of a false positive result of the BRAF mutation (Table 2 and Table 3).

The expression of all examined miRNAs was significantly higher in malignant lesions than benign lesions, both among AUS-nuclear and AUS-other nodules (Table 4). miR-146b expression was higher in PTCs than in other cancers (mean fold change values ± SD: 27.14 ± 43.6 vs. 1.98 ± 3.1, *p* = 0.0135, respectively), and, among PTCs, it was significantly higher in BRAF-positive than in BRAF-negative cases (38.5 ± 49.2 vs. 3.2 ± 4.4, *p* = 0.0190).

There were no such differences observed for other miRNAs (Table S2). For both AUS-nuclear and AUS-other nodules, the expression of miR-146b, -221 and -222 above the cut-off values determined by ROC curve analysis significantly increased the risk of their malignancy (Table 2 and Table 3). The cut-off value for miR-146b was two-times lower for AUS-other nodules than for AUS-nuclear nodules (0.7 vs. 1.6). For miR-222, the difference in the cut-off values was very small (0.8 vs. 0.7), and for miR-221, the thresholds were the same (0.2) for both types of nodules. The expression of miR-146b above the threshold was the only independent risk factor in both groups (AUS-nuclear, OR: 6.2, 95%CI: 1.2–33.2, *p* = 0.033; AUS-other, OR: 13.2, 95%CI: 1.6–106.5, *p* = 0.015).

**Figure 1:**
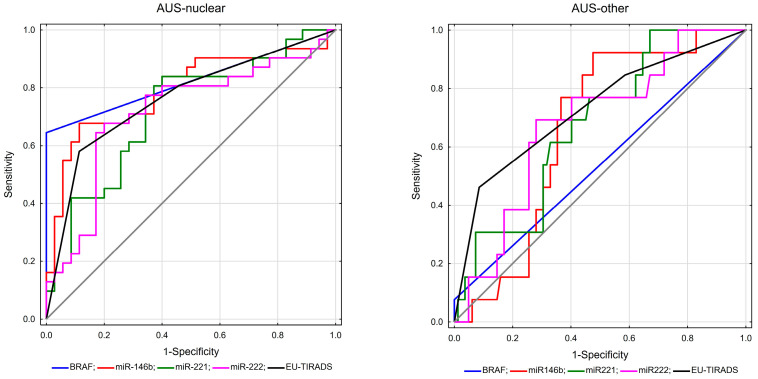
ROC curve analysis of the EU-TIRADS, BRAF mutation and miRNA evaluation in Bethesda category III nodules in classification with features of nuclear atypia (**AUS-nuclear**) or features of other types of atypia (**AUS-other**).

For all analyzed methods (EU-TIRADS, BRAF, miRNAs), the ability to differentiate benign lesions and cancers as measured by the area under the ROC curve was higher for AUS-nuclear nodules than for AUS-other nodules (Table 2 and Table 3). In the group of AUS-nuclear nodules, the AUC was the highest for the BRAF mutation, and in the group of AUS-other nodules, it was highest for the EU-TIRADS classification (Figure 1). However, the differences between the compared methods were not significant. Concerning miRNA expression in AUS-nuclear nodules, the highest AUC was obtained for miR-146b, while, in AUS-other nodules, it was observed for miR-222. Moreover, SPC was highest for these miRNAs (88.6% and 72.0%, respectively). SEN for both types of nodules was, in turn, highest for miR-221 (AUS-nuclear: 83.9%, AUS-other: 100.0). Unfortunately, it was accompanied by low SPC, especially among AUS-other nodules (32.9%). The expression of miR-221 above the established threshold, as the only evaluated method, identified all cases of cancer other than PTC (Table S3).

### 3.2. Efficiency of Examined Methods in rFNA-Negative Cancer Identification

Table 5 presents the results of the rFNA of the examined nodules. For both types of nodules (AUS-nuclear and AUS-other), rFNA most commonly brought the same diagnosis as the first cytological exam (AUS-nuclear: 53.0%; AUS-other: 53.7%). In the AUS-nuclear group, the rFNA outcome of category II BSRTC was less common than in the AUS-other group (12.1% vs. 35.8%, *p* = 0.0008). The malignancy rate in AUS-nuclear nodules of this rFNA category was 12.5%, and in AUS-other nodules, it was 2.9% (NS). The rFNA result was not positive in any case of AUS-other nodules. In the AUS-nuclear group, a positive rFNA outcome (category V or VI) was observed in 54.8% of cancers (17 out of 31); among them, there were 16 PTCs (including 15 BRAF-positive ones) and one squamous cell carcinoma. All of these cancers were also positive for EU-TIRADS categorization or BRAF mutation (Table 6). These two additional methods identified 77.4% (24 out of 31) cancers corresponding to AUS-nuclear nodules and 46.2% (6 out of 13) cancers corresponding to AUS-other nodules. All seven (100.0%) BRAF- and EU-TIRADS-negative cancers in the AUS-other group and six out of seven (85.7%) such cancers in the AUS-nuclear group could be revealed by a positive result of at least one miRNA. The assessment of EU-TIRADS, BRAF and miRNAs jointly could identify all 13 rFNA-negative cancers in the AUS-other group and 13 out of 14 (92.9%) cancers in the AUS-nuclear group. Tables S4 and S5 show detailed data on the efficiency of the examined methods in particular cancer cases in both groups.

### 3.3. Effectiveness of Combined Criteria Based on EU-TIRADS, BRAF Mutation and miRNA Assessment

The analysis of possible combined criteria based on BRAF, EU-TIRADS and miRNA analyses, without consideration of the rFNA outcome, showed that for AUS-nuclear nodules, the highest classification efficacy measured by ACC (84.8%) and LR+ (7.1) was obtained for the combination of BRAF positivity or miR-146b positivity (Table 7 and Table S6). These criteria showed 80.6% SEN and 88.6% SPC. The addition of miR-222 to this combination increased the SEN to 90.3%, with no change in ACC. The maximization of the SEN (96.8%) was ensured by the use of miR-221 instead of miR-222. However, an increase in SEN was accompanied by a significant decrease in SPC (to 60.0%) and an increase in the percentage of positive nodules (to 66.7%). The use of the rFNA outcome did not noticeably improve the combinations that showed the highest ACC (an increase of 1.6 percentage points) or the highest SEN (no increase).

In the case of AUS-other nodules, none of the possible combinations of the examined methods allowed for a simultaneous increase in SEN and SPC to a level above 80% (Table 7 and Table S7). SEN and SPC above 70% were obtained with the miR-222-positive or BRAF-positive combination (SEN: 76.9%; SPC: 72.0%); 34.7% of examined nodules met this criterion. The maximization of SEN (100.0%) with possibly high SPC (64.6%) was achieved for the association of EU-TIRADS with miR-222, with 44.2% of examined nodules meeting this criterion. Considering the BSRTC category II rFNA outcome for the sufficient exclusion of malignancy and applying the above additional criteria only to the remaining nodules was more effective. This approach increased the SPC of the classification and, with the EU-TIRADS-positive or miR-222-positive combined criterion, allowed us to achieve SEN of 92.3%, and it would have allowed us to forego surgical treatment in 67.4% of patients.

## 4. Discussion

The use of molecular tests to evaluate the material obtained during thyroid biopsy is the subject of continuous research. Commercially available molecular tests for thyroid FNAs that combine broad genotyping panels with gene and/or microRNA expression profiling are relatively expensive and usually require to perform an additional aspiration of the nodule or to split the routinely obtained material before making a smear [19,20]. Therefore, in our study, we decided to check whether a method free of these main disadvantages would be feasible in the case of nodules of category III in the Bethesda classification. To this end, we decided to use the material remaining in the needle after the preparation of the classic smear and to limit molecular diagnostics to a few well-recognized tests. Notably, at the same time, we assessed the potential of a combined ultrasound and molecular evaluation of nodules and considered the distinctiveness of the two main subcategories of category III: nodules with nuclear atypia and nodules with atypia of other types. As we have shown, this is a suitable approach that facilitates clinical decision making for both of these subcategories and prevents a significant number of diagnostic thyroid surgeries.

We confirmed our previous observations that among nodules with architectural atypia, rFNA is more effective in the reliable prediction of a nodule’s benignity, while, among nodules with nuclear atypia, it is effective in revealing malignancy [11]. When the rFNA result of a AUS-other nodule was classified as category II BSRTC, the risk of malignancy was reduced to approximately 3%, i.e., to the level characteristic of category II [4,5]. In the case of AUS-nuclear nodules in the equivalent situation, the risk of malignancy did not decrease satisfactorily and exceeded 12%. However, the rFNA of AUS-nuclear nodules allowed the detection of almost 55% of cancers (the rFNA result was classified as category V or VI BSRTC), while rFNA of AUS-other nodules was not effective in this aspect. This is not surprising because, in the case of PTCs, carcinomas typical of AUS-nuclear nodules, the diagnosis is determined by the microscopic features of cell nuclei that can be assessed in the FNA material. However, in the case of FTCs that more often correspond to AUS-other nodules, features that are important in confirming malignancy (e.g., infiltration of the nodule capsule) can be verified only in postoperative histopathological examination. Interestingly, we showed that all cancers revealed during the rFNA of AUS-nuclear nodules were also positive for EU-TIRADS (category 5) or BRAF mutations. In the case of other cancers (EU-TIRADS- and BRAF-negative), the miRNA assessment was highly effective. In our material, it allowed the identification of 92.9% of cancers negative for rFNA, EU-TIRADS and BRAF mutation, including all cancers corresponding to AUS-other nodules and 85.7% of cancers corresponding to AUS-nuclear nodules. This means that if the material for molecular tests had been preserved already during the first FNA, another biopsy could have been avoided with little risk of false negative results. However, subsequently, the problem would arise of a large percentage of false positive results, which characterizes the miRNA expression tests, especially in the case of miR-221 among AUS-other nodules. This problem can be at least partially overcome by the use of one or two other examined miRNAs combined with the assessment of the BRAF mutation or the EU-TIRADS category, i.e., tests with a very low (EU-TIRADS) or even zero (BRAF) false positive rate.

For AUS-nuclear nodules, the combined criteria that showed the highest ACC (84.8%) were based on two constant elements, the BRAF mutation and miR-146b expression. Adding miR-222 to this combination increased the SEN from 80.6% to 90.3% and potentially allowed nearly half (47%) of the patients to avoid diagnostic surgery. Such a set of tests had SPC of 80.0% with both SEN and NPV over 90%. Replacing miR-222 with miR-221 further increased the SEN to almost 97%, but lowered the SPC (to 60.0%) and allowed only one third of patients to avoid unnecessary surgery. The addition of the rFNA result to the combination did not further increase the maximal achieved SEN, nor did it change the set of tests leading to the highest ACC (BRAF/miR-146b), but it led to an increase in ACC and SEN by several percentage points in some combinations of miRNAs.

In the group of AUS-other nodules, the combination of miR-222 with EU-TIRADS achieved SEN of 100% with SPC of 64.6% and ACC of 69.5%. This set of tests would make it possible to safely avoid diagnostic surgery in 55.8% of the nodules. The use of a two-step procedure—at the first stage forgoing surgical treatment in subjects with a benign rFNA outcome and at the second stage the use of the above combined criterion (EU-TIRADS/miR-222) to assess nodules of categories I, III and IV in rFNA—increased the ACC and SPC by approximately 10 percentage points, with SEN still over 90% (92.3%), and would allow us to avoid surgery in 67.4% of patients. Nishino et al. [35] also pointed to an increase in SPC when molecular testing was limited to nodules with BSRTC III and IV outcomes of rFNA (they evaluated the commercial panel of Afirma GECy in material from BSRTC category III and IV nodules combined).

We also showed that in the case of AUS-nuclear nodules, which have a significantly higher risk of malignancy and a higher rate of PTCs among cancers, the diagnostic effectiveness of the examined ancillary methods was higher than in the case of AUS-other nodules. This was most evident in the assessment of the BRAF mutation, for which the AUC was clearly higher in the AUS-nuclear subcategory than in the AUS-other subcategory (0.823 vs. 0.538). We confirmed that the BRAF mutation test had SPC of 100% but insufficient SEN [36]. Its sensitivity, as analyzed for all category III nodules, was 54.5%, but it was many times lower among AUS-other nodules than AUS-nuclear nodules (7.7% vs. 64.5%). In the AUS-other subcategory, not only were PTCs rarer among cancers, but BRAF was also less likely to be positive (only 1/4 PTCs were BRAF-positive among AUS-other nodules vs. 3/4 among AUS-nuclear nodules). It can be assumed that BRAF-negative PTCs corresponded to follicular variant PTCs (fvPTCs), in which the presence of BRAF mutations is less frequent [37,38]. Unfortunately, we did not have information on the histopathologic subtype for all PTCs, and we could not perform such an analysis. However, this interpretation is supported by the fact that BRAF-negative PTCs were classified as the EU-TIRADS high-risk category less often than BRAF-positive ones (30.0% vs. 71.4%). There are numerous papers supporting our observations of the frequent co-occurrence of the BRAF mutation in nodules with high-risk features on US imaging [39] and reports indicating that ultrasound imaging of fvPTCs is less suggestive [13].

Differences in the diagnostic efficacy of BRAF mutation assessment between AUS-nuclear and AUS-other nodules are likely responsible for the significant discrepancies in opinions on the usefulness of this test in the diagnostics of category III nodules. Most of these analyses do not include information about the type of atypia dominating among the category III nodules in the examined material. In Asian countries such as Korea and Japan, with a long history of high iodine supply, cases with nuclear atypia among category III smears are twice as common as in non-Asian populations [7]. In consequence, the rate of malignancy is high in the Asian cohort, and the vast majority of cancers are PTCs [7]. Unsurprisingly, numerous publications indicating the validity of the assessment of BRAF mutations in nodules classified as category III come from these countries [14,40]. The situation is different in Western countries, especially those that have been exposed to iodine deficiency in the past. There, as in our population, benign nodules with architectural atypia predominate within category III (hyperplastic nodules and follicular adenomas) [41]. The assessment of BRAF mutation is obviously less effective in these circumstances, just as it is less effective in the case of category IV BSRTC, where the percentage of PTCs among cancers is often even lower [36,42,43,44,45]. Fnais et al. [46], in their meta-analysis, did not find any substantial evidence to support the implementation of BRAF mutation analysis as a single screening test for patients with indeterminate thyroid nodules (categories III–V together or separately in category III nodules). However, several authors agree that the combined assessment of the BRAF mutation and ultrasound risk can be effectively used in the diagnosis of BSRTC category III nodules [47,48]. Our analyses confirmed this opinion, but only with respect to AUS-nuclear nodules. We showed that in this subcategory, the combined use of BRAF mutation and EU-TIRADS assessment had higher SEN than each of these tests separately (BRAF or EU-TIRADS vs. EU-TIRADS and vs. BRAF, SEN: 77.4% vs. 58.1% vs. 64.5%, respectively). There was no similar effect among AUS-other nodules (SEN: 46.2% vs. 46.2% vs. 7.7%, respectively), but it is difficult to draw unambiguous conclusions in this matter due to the very rare occurrence of BRAF mutations among cancers in this subcategory in our material. The diagnostic efficacy of EU-TIRADS, as measured by the AUC, was only slightly lower among AUS-other nodules than AUS-nuclear nodules. This confirms previous observations of the system’s high versatility in relation to cancers other than PTCs [29,30]. What distinguishes EU-TIRADS from other similar systems is that EU-TIRADS category 5 includes not only taller-than-wide but also round nodules, and category 4 encompasses nodules with any hypoechoic part and not only dominating ones [1].

Regarding miRNA assessment, all examined miRNAs had higher diagnostic efficacy in AUS-nuclear nodules than in AUS-other ones, as measured with the AUC. As expected, the largest differences were observed for miR-146b, whose significant overexpression in PTCs has been confirmed by many researchers [27,32]. In our study, the ACC of the classification of AUS-nuclear nodules with miR-146b reached nearly 80%. The difference in the expression of miR-146b in PTCs and other cancers was reflected in a two-fold lower threshold for this miRNA in the AUS-other group compared to AUS-nuclear. In the case of AUS-other nodules, the best discriminatory ability among the examined miRNAs was for miR-222, which showed ACC of 71.6%. SEN scores for both types of nodules were highest for miR-221 (AUS-nuclear: 83.9%, AUS-other: 100.0%). Interestingly, it was found that the expression of miR-221 above the established threshold revealed all cases of cancer other than PTC and the largest fraction (80.0%) of BRAF-negative PTCs. It was therefore two-times more effective in the identification of BRAF-negative PTCs than assessing the EU-TIRADS category. This is consistent with the observations of other authors who found the overexpression of miR-221 and miR-222 in fvPTC, which is usually BRAF-negative [49]. Like us, Cai et al. [31] showed that miR-221 had higher sensitivity than miR-222 but lower specificity. Regardless of these differences, the expression of miR-146b, -221 and -222 above the cut-off values significantly increased the risk of malignancy in both groups of category III nodules.

Data on the association of the expression of the examined miRNAs with the presence of the BRAF mutation are inconclusive. In our study, we found higher expression of miR-146b in BRAF-positive than in BRAF-negative nodules and a similar but negligible trend for other miRNAs. Similar observations about miR-146b come from other reports [50,51], and for miR-221 and -222 from Sun et al. [50]. The association of miR-146b overexpression with the presence of the BRAF mutation is also indicated by its higher expression in classical variants of PTC than fvPTC or NIFTP [52]. On the other hand, Sheu et al. [49] showed no correlation between BRAF mutation and the expression of miR-146b, -221 and -222 in PTC subjects.

It should be emphasized that comparing data on the usefulness of molecular tests in the diagnostics of nodules with ambiguous cytology between centers is not easy [53]. This is due to several reasons. First, there is a significant difference between centers in the pre-test probability of the malignancy of the nodules as well as in the incidence of PTC among cancers. This is a consequence of differences in the criteria adopted in qualifying nodules for study (category III–V total vs. category III–IV total vs. separate assessment of each of these categories), epidemiological differences in the studied populations (primarily in terms of iodine supply) or the type of diagnostic center (basic vs. reference one, endocrine vs. oncological) [54]. Secondly, studies differ in the time of collecting material for molecular analysis: FNA ex vivo vs. material recovered from an archival smear, the first preoperative FNA vs. rFNA and, in the case of rFNA, the interpretation of its results. We consider category V BSRTC of rFNA outcomes to be sufficient to justify surgical treatment, but, in other centers, this can only apply to category VI. Controversy is also related to the interpretation of the diagnosis of benign lesions with rFNA [8,55,56,57]. Thirdly, some studies do not include histopathological verification in the case of the majority of benign molecular test results. There is also no consensus on how to classify low-risk thyroid neoplasms. Despite these discrepancies, most studies on non-commercial kits based on the evaluation of miRNAs alone or in combination with additional molecular markers conclude on their diagnostic usefulness. However, the proposed miRNA panels differ.

Nikiforova et al. [58] demonstrated that a set of miRNAs, including miR-187, miR-221, miR-222, miR-146b, miR-155, miR-224 and miR-197, could be diagnostically used with high accuracy to detect malignancies in consecutive thyroid FNA samples. Chen et al. [59] showed that miR-146b and miR-222 might be used as distinguishing markers for PTC in the material from FNA, but notably they evaluated FNA material obtained ex vivo and the diagnoses were based on surgical pathology reports. Laukienė et al. [60] examined nodules of Bethesda categories III–VI and found that the expression analysis of four miRNAs, miR-55a, miR-146b, miR-221 and miR-4324, improved the accuracy of FNA, but they did not analyze category III BSRTC separately. Keutgen et al. [61] evaluated FNA material obtained ex vivo from nodules classified into categories III–V BSRTC jointly and found that the expression of miR-222, miR-328, miR-197 and miR-21 combined in a predictive model was accurate in differentiating malignant from benign lesions. Castagna et al. [62], similarly to us, demonstrated that a diagnostic miRNA panel consisting of miR-146b, miR-221 and miR-222 could increase the diagnostic utility of FNA, but there were only seven nodules of categories III and IV BSRTC in their material, of which only one was verified with a postoperative histopathological examination. Kitano et al. [63] reported that miR-7 was a helpful adjunct marker to distinguish benign from malignant thyroid FNA samples (FNA performed intraoperatively, nodules of category III constituted less than 20% of the total). Santos et al. [64,65] created a panel consisting of 11 miRNAs, including let-7a, miR-103, miR-125a-5p, let-7b, miR-145, RNU48, miR-146b, miR-152, miR-155, miR-200b and miR-181, and proved its diagnostic utility in differentiating between benign and malignant category III–V nodules and for a reduction in the number of unnecessary surgeries. They evaluated material obtained from archival smears and reported ACC of 83.3% in a set of 18 nodules of category III (including one cancer), but, in another set of 92 similar nodules (including 52 cancers), the ACC was only 66.3%. Shen et al. [66] assessed archival material obtained from the FNA of nodules of various BSRTC categories (including category III) and found that a set of four miRNAs, miR-146b, miR-221, miR-187 and miR-30d, could differentiate PTCs from benign lesions, but it had low accuracy in classifying ‘atypia of undetermined significance’ when it corresponded to follicular neoplasia or fvPTC. Mazeh et al. [67] evaluated a panel consisting of 19 miRNAs, miR-146b, miR-146, miR-222, miR-221, miR-134, miR-34a, miR-101, miR-143, miR-144, miR-615, miR-375, miR-181b, miR-194, miR-130a, miR-199a-3p, miR-30a, miR-424, miR-148a and miR-24, in a group of nodules with indeterminate FNA (categories III and IV jointly) and they reported its high effectiveness, but they performed FNA ex vivo and all thyroid malignancies were PTCs. Agretti et al. [44] found that the expression profiles of three miRNAs, miR-146b, miR-155 and miR-221, allowed them to distinguish benign nodules from PTCs, but the panel was not effective in the group of indeterminate lesions (they examined nodules of category IV BSRTC) due to low sensitivity and specificity. Paskas et al. [68] demonstrated a reduction in the number of individuals undergoing surgery by half through the use of the assessment of the BRAF mutation, miR-221, miR-222 and galectin-3 in FNA samples of category III–V BSRTC. Stokowy et al. [69] have suggested that two miRNA markers (miR-484, miR-148b-3p) might improve the classification of mutation-negative FTCs and adenomas (they analyzed freshly frozen samples). Panebianco et al. [70] have proposed the use of a panel of two miRNAs, miR-146b and miR-222 (both included in our study), and two genes (KIT and TC1) as a further step in the diagnosis of nodules with indeterminate cytology and a wild-type BRAF variant. Their study included FNA material from 12 nodules described as ‘indeterminate follicular proliferation’. None of the above papers evaluated nodules of category III with nuclear atypia or with architectural atypia separately. In this respect, our work is the first study of this type.

The limitations of our study include patient selection bias that was a consequence of the exclusion of all patients who did not undergo thyroid surgery. On the other hand, this approach increases the reliability of the final diagnoses. Despite this exclusion, the incidence of AUS-nuclear and AUS-other nodules in the analyzed material and the frequency of PTC among cancers (both approximately three-times higher for AUS-nuclear than AUS-other nodules) were consistent with those previously observed by us in a larger group of subsequent patients with such nodules [11]. The low percentage of cancers among AUS-other nodules in the assessed population means that, especially in this subcategory, the results obtained by us need to be verified on a larger number of nodules. Another limitation of our study is the lack of a detailed assessment of PTC subtypes in all cases.

## 5. Conclusions

**Figure A1:**
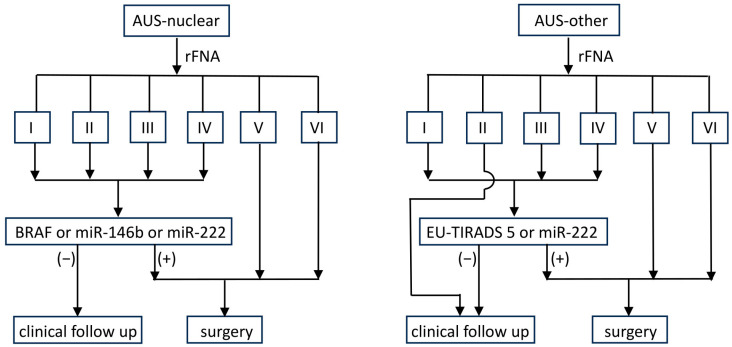
Proposed algorithm for clinical management of category III nodules with the use of molecular tests with respect to the Bethesda category of repeat FNA (rFNA) outcome.

In conclusion, the evaluation of three miRNAs with recognized diagnostic potential for the differentiation of thyroid nodules, in combination with BRAF mutation and EU-TIRADS category assessment, supports clinical decision making in patients with thyroid nodules of category III. The optimal use of these methods, however, requires a separate approach to nodules with features of nuclear atypia and nodules with features of architectural atypia. These nodules differ significantly in the pre-test probability of malignancy and the frequency of PTC among cancers. For nodules with features of architectural atypia, the most effective diagnostic panel includes the assessment of miR-222 and the EU-TIRADS category. It is possible to limit molecular testing only to AUS-other nodules with a non-diagnostic or indeterminate rFNA result (defined as BSRTC categories III and IV). This is associated with SEN of 92.3% and SPC of 76.8%. In the case of AUS-nuclear nodules, the set of tests recommended by us includes, in addition to rFNA, determination of the BRAF mutation and the expression of miR-146b and miR-222. Such a set of tests allows us to achieve SEN of 93.5% and SPC of 80.0%. The optimal diagnostic algorithm for nodules of category III is presented in the Appendix A (Figure A1). The test sets proposed by us have, according to the analyses of Vargas-Salas et al. [22], the characteristics of ‘rule-out’ tests in the case of AUS-other nodules and the characteristics of ‘rule-in’ tests in the case of AUS-nuclear nodules. However, both sets can function well as both rule-out and rule-in tests owing to SEN at the level of 90% and SPC close to 80%.

## Figures and Tables

**Table 1:** Characteristics of examined nodules of category III in the Bethesda classification that presented features of nuclear atypia (AUS-nuclear) or features of other atypia (AUS-other).

	AUS-Nuclear	AUS-Other	*p*
**No. of nodules**	66	95	
**No. of patients**	62	88	
**No./% of males among patients**	12/19.4	10/11.4	0.1731
**Mean age of patients ± SD [year]**	58.4 ± 14.5	55.6 ± 14.4	0.2354
**Mean volume of nodules ± SD [cm^3^]**	5.8 ± 20.5	4.4 ± 8.9	0.5786
**No./% of benign nodules**	35/53.0	82/86.3	<0.0001
**No./% of cancers**	31/47.0	13/13.7	
**No./% of papillary carcinomas among cancers**	27/87.1	4/30.8	0.0007
**No. of follicular carcinomas**	1	3	
**No. of non-follicular cell-derived cancers**	1	3	
**No. of follicular cell-derived low-risk neoplasms**	2	3	

**Table 2:** Diagnostic efficiency of the assessment of EU-TIRADS category, BRAF mutation and miRNAs (146b, 221 and 222) in the diagnostics of Bethesda category III nodules with features of nuclear atypia (AUS-nuclear).

	EU-TIRADS	BRAF	miR-146b	miR-221	miR-222
**AUC (95%CI)** ***p***	0.761 (0.642-0.880) *p* = 0.0011	0.823 (0.713–0.932) *p* < 0.0001	0.790 (0.674–0.905) *p* < 0.0001	0.720 (0.595–0.845) *p* = 0.0006	0.716 (0.586–0.846) *p* = 0.0011
**Threshold**	5	-	1.6	0.2	0.7
**TP [No./%]**	18/27.3	20/30.3	21/31.8	26/39.4	21/31.8
**FP [No./%]**	4/6.1	0/0.0	4/6.1	14/21.2	7/10.6
**FN [No./%]**	13/19.7	11/16.7	10/15.2	5/7.6	10/15.2
**TN [No./%]**	31/47.0	35/53.0	31/47.0	21/31.8	28/42.4
**SEN [%]**	58.1	64.5	67.7	83.9	67.7
**SPC [%]**	88.6	100.0	88.6	60.0	80.0
**ACC [%]**	74.2	83.3	78.8	71.2	74.2
**PPV [%]**	81.8	100.0	84.0	65.0	75.0
**NPV [%]**	70.5	76.1	75.6	80.8	73.7
**LR+**	5.1	-	5.9	2.1	3.4
**No./% of nodules**	22/33.3	20/30.3	25/37.9	40/60.6	28/42.4
**OR (95%CI)** ***p***	10.7 (3.0–37.9) 0.0002	>100 (0.0–) 0.9969	16.3 (4.5–58.8) <0.0001	7.8 (2.4–25.2) 0.0006	8.4 (2.7–25.7) 0.0002

**Table 3:** Diagnostic efficiency of the assessment of EU-TIRADS category, BRAF mutation and miRNAs (146b, 221 and 222) in the diagnostics of Bethesda category III nodules with features of atypia other than nuclear (AUS-other).

	EU-TIRADS	BRAF	miR-146b	miR-221	miR-222
**AUC (95%CI)** ***p***	0.729 (0.569–0.889) *p* = 0.0049	0.538 (0.361–0.716) *p* = 0.6715	0.657 (0.532–0.783) *p* = 0.0140	0.674 (0.535–0.814) *p* = 0.0139	0.678 (0.533–0.823) *p* = 0.0162
**Threshold**	5	-	0.7	0.2	0.8
**TP [No./%]**	6/6.3	1/1.1	12/12.6	13/13.7	9/9.5
**FP [No./%]**	7/7.4	0/0.0	39/41.1	55/57.9	23/24.2
**FN [No./%]**	7/7.4	12/12.6	1/1.1	0/0.0	4/4.2
**TN [No./%]**	75/78.9	82/86.3	43/45.3	27/28.4	59/62.1
**SEN [%]**	46.2	7.7	92.3	100.0	69.2
**SPC [%]**	91.5	100.0	52.4	32.9	72.0
**ACC [%]**	85.3	87.4	57.9	42.1	71.6
**PPV [%]**	46.2	100.0	23.5	19.1	28.1
**NPV [%]**	91.5	87.2	97.7	100.0	93.7
**LR+**	5.4	-	1.9	1.5	2.5
**No./% of nodules**	13/13.7	1/1.1	51/53.7	68/71.6	32/33.7
**OR (95%CI)** ***p***	9.2 (2.4–34.9) 0.0012	>100 (0.0–) 0.9972	13.2 (1.6–106.5) 0.0152	-	5.8 (1.6–20.6) 0.0069

**Table 4:** Expression of examined miRNAs in benign and malignant category III nodules with features of nuclear atypia (AUS-nuclear) or features of atypia of other type (AUS-other).

Fold Change Value	miR-146b		miR-221		miR-222	
	Benign Nodules	Malignant Nodules	Benign Nodules	Malignant Nodules	Benign Nodules	Malignant Nodules
**AUS-nuclear**						
**Mean ± SD**	2.83 ± 8.6	27.4 ± 43.5	0.57 ± 1.0	2.7 ± 7.7	0.69 ± 1.1	2.69 ± 4.8
**Median** **(Q25–Q75)**	0.46 (0.23–1.42)	10.6 (0.74–29.4)	0.13 (0.07–0.84)	0.77 (0.28–1.90)	0.23 (0.12–0.65)	0.94 (0.46–2.02)
***p***	<0.0001		0.0022		0.0026	
**AUS-other**						
**Mean ± SD**	1.17 ± 1.5	1.34 ± 0.8	0.89 ± 1.4	1.80 ± 2.2	0.84 ± 1.5	1.09 ± 0.9
**Median** **(Q25–Q75)**	0.63 (0.24–1.59)	1.13 (0.92–1.57)	0.43 (0.16–0.97)	0.86 (0.47–2.86)	0.38 (0.18–0.87)	0.83 (0.49–1.15)
***p***	0.0497		0.0434		0.0412	

**Table 5:** Outcomes of repeat FNA of category III nodules with features of nuclear atypia (AUS-nuclear) or features of atypia of other type (AUS-other).

Definite Diagnosis	Category in the Bethesda System [No./%]						
	I	II	III AUS-Other	III AUS-Nuclear	IV	V	VI
**AUS-nuclear**							
**Malignant**	0	1/3.2	1/3.2	12/38.7	0	5/16.1	12/38.7
**Benign**	1/2.9	7/20.0	4/11.4	23/65.7	0	0	0
**AUS-other**							
**Malignant**	0	1/7.7	10/76.9	0	2/15.4	0	0
**Benign**	7/8.5	33/40.2	41/50.0	0	1/1.2	0	0

**Table 6:** Efficiency of examined methods in identification of cancer in relation to repeat FNA (rFNA) status (positive/negative) in group with nuclear atypia (AUS-nuclear) and group with atypia of other type (AUS-other).

rFNA Result in Cancer	No. of Positive Cases						
	EU-TIRADS	BRAF	miR-146b	miR-221	miR-222	EU-TIRADS or BRAF	EU-TIRADS or BRAF or miRNAs
**AUS-nuclear**							
**Positive—17**	12	15	14	16	13	17	17
**Negative—** **14**	6	5	7	10	8	7	13
**AUS-other**							
**Positive—** **0**	-	-	-	-	-	-	-
**Negative—** **13**	6	1	12	13	9	6	13

**Table 7:** Best joint criteria based on the evaluation of miRNAs, EU-TIRADS and BRAF mutation that differentiate benign from malignant lesions in the group of nodules with nuclear atypia (AUS-nuclear) and group of nodules with other atypia (AUS-other).

Criteria	SEN [%]	SPC [%]	ACC [%]	PPV [%]	NPV [%]	LR+	% of Nodules
**AUS-nuclear**							
BRAF/miR-146b	80.6	88.6	84.8	86.2	83.8	7.1	43.9
BRAF/miR-146b/miR-222	90.3	80.0	84.8	80.0	90.3	4.5	53.0
BRAF/miR-146b/miR-221	96.8	60.0	77.3	68.2	95.5	2.4	66.7
BRAF/miR-146b/rFNA	83.9	88.6	86.4	86.7	86.1	7.3	45.5
BRAF/miR-146b/miR-222/rFNA	93.5	80.0	86.4	80.6	93.3	4.7	54.5
BRAF/miR-146b/miR-221 /rFNA	96.8	60.0	77.3	68.2	95.5	2.4	66.7
**AUS-other**							
BRAF/miR-222	76.9	72.0	72.6	30.3	95.2	2.7	34.7
EU-TIRADS/miR-222	100.0	64.6	69.5	31.0	100.0	2.8	44.2
**AUS-other, nodules of category II in rFNA regarded as benign and excluded**							
BRAF/miR-222	76.9	84.1	83.2	43.5	95.8	4.9	24.2
EU-TIRADS/miR-222	92.3	76.8	78.9	38.7	98.4	4.0	32.6

## Data Availability

The data presented in this study are available on request from the corresponding author. The data are not publicly available due to patient privacy restrictions.

## Supplementary Materials

The following supporting information can be downloaded at: https://www.mdpi.com/article/10.3390/cancers15174287/s1, Table S1: Classification of benign and malignant Bethesda category III nodules with features of nuclear atypia (AUS-nuclear) or features of other atypia (AUS-other) into particular EU-TIRADS categories; Table S2: Expression of examined miRNAs in papillary carcinoma (PTC), follicular carcinoma (FTC), cancers derived from cells other than thyroid follicular cells and follicular cell-derived low-risk neoplasms; Table S3: Efficiency of EU-TIRADS, BRAF and miRNA (miR-146b, -221, -222) assessment in diagnosing particular types of cancer; Table S4: Status of EU-TIRADS (category 5), BRAF mutation, overexpression of miR-146b, -221, -222 and rFNA (Bethesda category V or VI) in particular cases of cancer identified in Bethesda category III nodules with features of nuclear atypia (AUS-nuclear); Table S5: Status of EU-TIRADS (category 5), BRAF mutation, overexpression of miR-146b, -221, -222 and rFNA (Bethesda category V or VI) in particular cases of cancer identified in Bethesda category III nodules with features of atypia other than nuclear (AUS-other); Table S6: Diagnostic efficiency of joint criteria based on the expression of miRNAs 146b, 221 and 222, EU-TIRADS category and the BRAF mutation in the group of Bethesda category III nodules with features of nuclear atypia (AUS-nuclear); Table S7: Diagnostic efficiency of joint criteria based on the expression of miRNAs 146b, 221 and 222, EU-TIRADS category and the BRAF mutation in the in the group of Bethesda category III nodules with features of atypia other than nuclear (AUS-other).

## Appendix A

The obtained results encouraged us to propose an algorithm for the clinical management of category III nodules with the use of molecular tests and the EU-TIRADS classification. According to this algorithm, all category III nodules are subjected to repeat FNA. The outcome of categories V or VI BSRTC mandates thyroid surgery. The outcome of category II in the case of nodules with atypia other than the nuclear type (AUS-other) allows for a conservative approach without further immediate testing, while all other outcomes should be followed by molecular tests. In the case of nodules with nuclear atypia (AUS-nuclear), these tests consist of BRAF V600E mutation and the expression of miR-146b and miR-222. Overexpression of any miRNA or the presence of the BRAF mutation warrants surgical treatment. In the case of AUS-other nodules, all nodules of EU-TIRADS category 5 should be treated surgically without molecular testing, and nodules of lower categories should be examined for the expression of miR-222. Its overexpression warrants surgery. In every case, when surgery is warranted, the extent of resection, or, in some cases, close surveillance instead of surgery, should be determined individually.
