# Supplementary material for: Effective Use of microRNA, BRAF and Sonographic Risk Assessment in Bethesda III Thyroid Nodules Requires a Different Approach to Nodules with Features of Nuclear Atypia and Other Types of Atypia
Source: Cancers (Basel). 2023 Aug 27;15(17):4287. doi: 10.3390/cancers15174287 (PMC10486535; doi:10.3390/cancers15174287)
Supplement: Supplementary file 1 [file cancers-15-04287-s001.zip › cancers-2572202-supplementary.pdf]

Table S1. Classification of benign and malignant Bethesda category III nodules with features of nuclear atypia (AUS-nuclear) or features of other atypia (AUS-other) into particular EU-TIRADS categories.

	AUS-nuclear		AUS-other	
	**benign nodules**	**malignant nodules**	**benign nodules**	**malignant nodules**
**EU-TIRADS 3 [No./%]**	19/54.3 a	6/19.4	34/41.5	2/15.4
**EU-TIRADS 4 [No./%]**	12/34.3	7/22.6	41/50.0	5/38.5
**EU-TIRADS 5 [No./%]**	4/11.4 b	18/58.1	7/8.5 c	6/46.2

a – p=0.0025 vs malignant nodules; b – p=0.0001 vs malignant nodules; c – p=0.0002 vs malignant nodules

Table S2. Expression of examined miRNAs in papillary carcinoma (PTC), follicular carcinoma (FTC), cancers derived from cells other than thyroid follicular cells and follicular cell–derived low-risk neoplasms.

**fold change value**	**PTC**	**FTC**	**Non follicular cell-derived cancers**	**Follicular cell–derived low-risk neoplasms**
		**miR-146b**		
**mean ± SD**	27.14±43.6	1.04±0.3	3.52±5.7	1.49±1.1
**median**	8.07	1.15	0.91	1.13
**(Q25-Q75)**	(1.01-29.42)	(0.81-1.28)	(0.46-6.58)	(0.92-1.40)
		**miR-221**		
**mean ± SD**	2.90±7.7	1.2±1.1	0.75±0.2	1.9±2.9
**median**	0.89	0.72	0.83	0.76
**(Q25-Q75)**	(0.26-3.17)	(0.55-1.86)	(0.63-0.87)	(0.37-0.77)
		**miR-222**		
**mean ± SD**	2.71±4.8	0.89±0.3	1.05±0.7	1.15±1.1
**median**	1.05	0.98	0.84	0.82
**(Q25-Q75)**	(0.18-2.37)	(0.64-1.14)	(0.66-1.43)	(0.78-0.84)

p=0.0135 - expression of miR-146b in PTC vs other cancers together

Table S3. Efficiency of EU-TIRADS, BRAF and miRNAs (miR-146b, -221, -222) assessment in diagnosing particular types of cancer.

Type of cancer	No of cases	number of identified cancers				
		EU-TIRADS	BRAF	miR-146b	miR-221	miR-222
**AUS-nuclear**						
**Papillary carcinoma**	27	16	20	20	22	18
**Follicular carcinoma**	1	0	0	0	1	0
**Non follicular cell-derived cancers**	1	1	0	1	1	1
**Follicular cell–derived low-risk neoplasms**	2	1	0	0	2	2
**AUS-other**						
**Papillary carcinoma**	4	2	1	4	4	2
**Follicular carcinoma**	3	1	0	3	3	3
**Non follicular cell-derived cancers**	3	2	0	2	3	2
**Follicular cell–derived low-risk neoplasms**	3	1	0	3	3	2

Table S4. Status of EU-TIRADS (category 5), BRAF mutation, overexpression of miR-146b, -221, -222, and rFNA (Bethesda category V or VI) in particular cases of cancers identified in Bethesda category III nodules with features of nuclear atypia (AUS-nuclear).

Type of cancer	EU-TIRADS	BRAF	miR -146b	miR -221	miR -222	rFNA
papillary carcinoma	+	+	+	+	+	+
papillary carcinoma	+	+	+	+	+	+
papillary carcinoma	+	+	+	+	+	+
papillary carcinoma	+	+	+	+	+	+
papillary carcinoma	+	+	+	+	+	+
papillary carcinoma	+	+	+	+	+	+
papillary carcinoma	+	+	+	+	+	+
papillary carcinoma	+	+	+	+	+	+
papillary carcinoma	+	+	+	+	+	-
papillary carcinoma	+	+	+	-	-	-
papillary carcinoma	+	+	-	-	-	-
papillary carcinoma	+	+	-	+	-	+
papillary carcinoma	+	+	-	+	-	-
papillary carcinoma	+	+	-	-	-	+
squamous cell carcinoma	+	-	+	+	+	+
papillary carcinoma	+	-	+	+	+	-
non-invasive follicular thyroid neoplasm with papillary-like nuclear features	+	-	-	+	+	-
papillary carcinoma	+	-	-	+	-	+
papillary carcinoma	-	+	+	+	+	+
papillary carcinoma	-	+	+	+	+	+
papillary carcinoma	-	+	+	+	+	+
papillary carcinoma	-	+	+	+	+	+
papillary carcinoma	-	+	+	+	+	-
papillary carcinoma	-	+	+	+	-	+
papillary carcinoma	-	-	+	+	+	-
papillary carcinoma	-	-	+	+	+	-
papillary carcinoma	-	-	+	-	-	-
papillary carcinoma	-	-	-	+	+	-
hyalinizing trabecular tumor	-	-	-	+	+	-
follicular carcinoma	-	-	-	+	-	-
papillary carcinoma	-	-	-	-	-	-

Table S5. Status of EU-TIRADS (category 5), BRAF mutation, overexpression of miR-146b, -221, -222, and rFNA (Bethesda category V or VI) in particular cases of cancers identified in Bethesda category III nodules with features of atypia other than nuclear (AUS-other).

Type of cancer	EU-TIRADS	BRAF	miR -146b	miR -221	miR -222	rFNA
papillary carcinoma	+	+	+	+	-	-
follicular carcinoma	+	-	+	+	+	-
papillary carcinoma	+	-	+	+	-	-
malignant lymphoma	+	-	+	+	-	-
thyroid tumor of uncertain malignant potential	+	-	+	+	-	-
medullary carcinoma	+	-	-	+	+	-
follicular carcinoma	-	-	+	+	+	-
secondary tumor	-	-	+	+	+	-
thyroid tumor of uncertain malignant potential	-	-	+	+	+	-
follicular carcinoma	-	-	+	+	+	-
papillary carcinoma	-	-	+	+	+	-
thyroid tumor of uncertain malignant potential	-	-	+	+	+	-
papillary carcinoma	-	-	+	+	+	-

Table S6. Diagnostic efficiency of joint criteria based on the expression of miRNAs 146b, 221 and 222, EU-TIRADS category and the BRAF mutation in the group of Bethesda category III nodules with features of nuclear atypia (AUS-nuclear).

Criterion	AUS-nuclear						
	SEN	SPC	ACC	PPV	NPV	LR+	% of nodules
miR-146b or -221	90.3	60.0	74.2	66.7	87.5	2.3	63.6
miR-146b or -222	77.4	80.0	78.8	77.4	80.0	3.9	47.0
miR-221 or -222	83.9	57.1	69.7	63.4	80.0	2.0	62.1
any miRNA	90.3	57.1	72.7	65.1	87.0	2.1	65.2
BRAF or miR-146b	80.6	88.6	84.8	86.2	83.8	7.1	43.9
BRAF or miR-221	93.5	60.0	75.8	67.4	91.3	2.4	65.2
BRAF or miR-222	87.1	80.0	83.3	79.4	87.5	4.4	51.5
BRAF or miR-146b or -221	96.8	60.0	77.3	68.2	95.5	2.4	66.7
BRAF or miR-146b or -222	90.3	80.0	84.8	80.0	90.3	4.5	53.0
BRAF or miR-221 or -222	93.5	57.1	74.2	65.9	90.9	2.2	66.7
BRAF or any miRNA	96.8	57.1	75.8	66.7	95.2	2.3	68.2
EU-TIRADS or miR-146b	87.1	77.1	81.8	77.1	87.1	3.8	53.0
EU-TIRADS or miR-221	93.5	51.4	71.2	63.0	90.0	1.9	69.7
EU-TIRADS or miR-222	87.1	68.6	77.3	71.1	85.7	2.8	57.6
EU-TIRADS or miR-146b or -221	96.8	51.4	72.7	63.8	94.7	2.0	71.2
EU-TIRADS or miR-146b or -222	93.5	68.6	80.3	72.5	92.3	3.0	60.6
EU-TIRADS or miR-221 or -222	93.5	48.6	69.7	61.7	89.5	1.8	71.2
EU-TIRADS or any miRNA	96.8	48.6	71.2	62.5	94.4	1.9	72.7
BRAF or EU-TIRADS	77.4	88.6	83.3	85.7	81.6	6.8	42.4
BRAF or EU-TIRADS or miR-146b	87.1	77.1	81.8	77.1	87.1	3.8	53.0
BRAF or EU-TIRADS or miR-221	93.5	51.4	71.2	63.0	90.0	1.9	69.7
BRAF or EU-TIRADS or miR-222	90.3	68.6	78.8	71.8	88.9	2.9	59.1
BRAF or EU-TIRADS or miR-146b or -221	96.8	51.4	72.7	63.8	94.7	2.0	71.2
BRAF or EU-TIRADS or miR-146b or -222	93.5	68.6	80.3	72.5	92.3	3.0	60.6
BRAF or EU-TIRADS or miR-221 or -222	93.5	48.6	69.7	61.7	89.5	1.8	71.2
BRAF or EU-TIRADS or any miRNA	96.8	48.6	71.2	62.5	94.4	1.9	72.7

Table S7. Diagnostic efficiency of joint criteria based on the expression of miRNAs 146b, 221 and 222, EU-TIRADS category and the BRAF mutation in the in the group of Bethesda category III nodules with features of atypia other than nuclear (AUS-other).

Criterion	AUS-other						
	**SEN**	**SPC**	**ACC**	**PPV**	**NPV**	**LR+**	**% of nodules**
miR-146b or -221	100.0	30.5	40.0	18.6	100.0	1.4	73.7
miR-146b or 222	100.0	51.2	57.9	24.5	100.0	2.1	55.8
miR-221 or 222	100.0	32.9	42.1	19.1	100.0	1.5	71.6
any miRNA	100.0	30.5	40.0	18.6	100.0	1.4	73.7
BRAF or miR-146b	92.3	52.4	57.9	23.5	97.7	1.9	53.7
BRAF or miR-221	100.0	32.9	42.1	19.1	100.0	1.5	71.6
BRAF or miR-222	76.9	72.0	72.6	30.3	95.2	2.7	34.7
BRAF or miR-146b or -221	100.0	30.5	40.0	18.6	100.0	1.4	73.7
BRAF or miR-146b or -222	100.0	51.2	57.9	24.5	100.0	2.1	55.8
BRAF or miR-221 or -222	100.0	32.9	42.1	19.1	100.0	1.5	71.6
BRAF or any miRNA	100.0	30.5	40.0	18.6	100.0	1.4	73.7
EU-TIRADS or miR-146b	100.0	46.3	53.7	22.8	100.0	1.9	60.0
EU-TIRADS or miR-221	100.0	30.5	40.0	18.6	100.0	1.4	73.7
EU-TIRADS or miR-222	100.0	64.6	69.5	31.0	100.0	2.8	44.2
EU-TIRADS or miR-146b or -221	100.0	28.0	37.9	18.1	100.0	1.4	75.8
EU-TIRADS or miR-146b or -222	100.0	45.1	52.6	22.4	100.0	1.8	61.1
EU-TIRADS or miR-221 or -222	100.0	30.5	40.0	18.6	100.0	1.4	73.7
EU-TIRADS or any miRNA	100.0	28.0	37.9	18.1	100.0	1.4	75.8
BRAF or EU-TIRADS	46.2	91.5	85.3	46.2	91.5	5.4	13.7
BRAF or EU-TIRADS or miR-146b	100.0	46.3	53.7	22.8	100.0	1.9	60.0
BRAF or EU-TIRADS or miR-221	100.0	30.5	40.0	18.6	100.0	1.4	73.7
BRAF or EU-TIRADS or miR-222	100.0	64.6	69.5	31.0	100.0	2.8	44.2
BRAF or EU-TIRADS or miR-146b or -221	100.0	28.0	37.9	18.1	100.0	1.4	75.8
BRAF or EU-TIRADS or miR-146b or -222	100.0	45.1	52.6	22.4	100.0	1.8	61.1
BRAF or EU-TIRADS or miR-221 or -222	100.0	30.5	40.0	18.6	100.0	1.4	73.7
BRAF or EU-TIRADS or any miRNA	100.0	28.0	37.9	18.1	100.0	1.4	75.8
